# Comparative study of gait parameters of patients undergoing distal femoral resections with non-operated and healthy limbs: a meta-analysis study

**DOI:** 10.3389/fonc.2023.1089609

**Published:** 2023-09-21

**Authors:** Nishant Banskota, Xiang Fang, Dechao Yuan, Wenli Zhang, Hong Duan

**Affiliations:** Department of Orthopedics, Orthopedic Research Institute, West China Hospital, Sichuan University, Chengdu, China

**Keywords:** distal femur tumor, gait analysis, stance phase, swing phase, cadence, velocity

## Abstract

**Introduction:**

Gait analysis is one of the most important components of functional outcome evaluation in patients with lower-extremity tumors. Disparities between operated limbs when compared with non-operated limbs and healthy populations based on gait parameters have rarely been studied. In the present study, we attempted to analyze the gait difference and its impacts on daily life.

**Methods:**

The gait parameters of distal femoral tumor-resected patients were collected from PubMed, CNKI, MEDLINE, Embase, Cochrane, and Google Scholar till September 30, 2022, by strictly following the inclusion and exclusion criteria. Differences between gait parameters in the operated and non-operated limbs or healthy limbs of distal femoral tumor patients were analyzed based on stance phase, swing phase, cadence, and velocity. The fixed-effects and random-effects models were used to conduct a meta-analysis.

**Results:**

Six studies were included according to the selection criteria. There were 224 patients in total in these studies. Standard mean differences were calculated for all of our outcomes. Our results showed that there was a minimal difference in the standard mean difference of gait parameters between operated and non-operated limbs and healthy limbs.

**Conclusion:**

Distal femoral tumor resections have been associated with deficient muscle function and strength and impaired gait parameters. Minimal differences in the gait parameters highlighted the advantage of distal femoral resection when replaced with a prosthesis.

## Introduction

The distal femur is a common site for primary bone neoplasm, and metastatic neoplasm lesions are common at the proximal femur (1). The appropriate option for reconstruction of the lower limb after resection of the femur or tibia is controversial (2). Options include the use of autografts (3), allografts (4), custom-made mega prostheses (5), and modular endoprostheses. The functional outcome for patients treated with distal femoral replacement prosthesis generally is thought to be acceptable. Before the introduction of this prosthesis, amputation was the preferred choice of treatment for these lesions (2). These custom-made prostheses are associated with lower complications but with increased costs/expenses (2). Restoration of gait function after reconstruction has been associated with improved functional outcomes and increased longevity of the reconstruction (6). Only a few studies have been conducted on gait analysis in patients after musculoskeletal tumor resection and reconstruction of the lower limb (7).

Gait analysis, which was founded by Jacquelin Perry, has been widely used in different fields of orthopedics (8). In gait analysis, the gait pattern is analyzed and studied in two different approaches based on non-wearable sensors (9). Gait is a highly valuable function that represents the integration of various physiological systems, including the central and peripheral nervous systems, perceptual system, and musculoskeletal system (10). Non-wearable systems (NWSs) analyze gait in the laboratory; in contrast, wearable systems (WSs) analyze gait data outside the laboratory during the person’s everyday activities (9). Walking movements are controlled by the structures of the lower limb, mainly the ankle and foot joints. The axis of rotation with each joint allows for joints to have a predominant plane of motion, perpendicular to that axis (11). Several studies stated that the gait cycle is significantly changed in walking velocity and stride length in patients undergoing prosthetic replacement of the distal femur, in comparison with the normal population (7, 12). These changes in walking need to be studied, as the quality of life counts are valued more by tumor patients due to their shortened life span (10).

By searching abundant pieces of literature on gait analysis and distal femoral tumor resection, we conducted this meta-analysis to obtain a comprehensive conclusion on gait parameters in distal femoral tumor patients treated with resections. These results will help us to guide and manage distal femoral tumor patients and improve their gait parameters. In our study, we compared gait parameters, mainly temporal–spatial parameters such as the stance phase, cadence, and velocity along with the swing phase in the operated limbs versus the non-operated limbs. The operated limbs were set as the experimental group, and the non-operated limbs and healthy limbs were set as the control group.

## Methods

**Figure 1:**
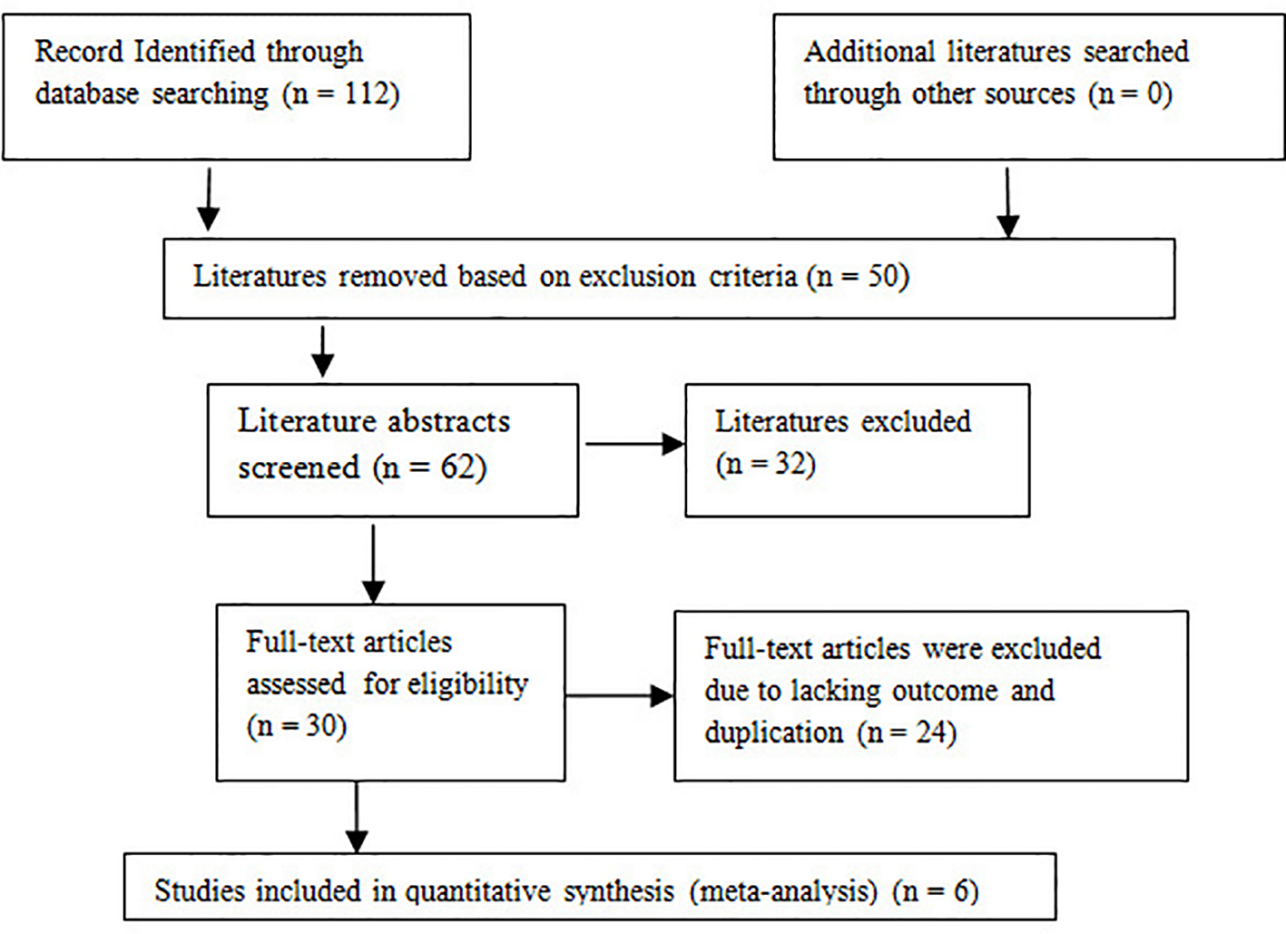
Flowchart of studies included and excluded.

Preferred Reporting Items for Systematic Reviews and Meta-Analyses (PRISMA) guidelines were followed to perform this study (**Figure 1**) (13).

### Literature search

PubMed, CNKI, MEDLINE, Cochrane, Embase, and Google Scholar databases were searched for relevant data till September 30, 2022. Relevant article searching started on October 1, 2021, and three authors of our study used 1-year duration. The reference lists of relevant studies were also hand-searched by two different authors. Keywords used for searching included distal femoral tumor resection, gait analysis, stance phase, swing phase, cadence, and velocity. Also, a manual search of the reference lists of the studies found in the databases was conducted.

### Included studies

#### Inclusion criteria

1) English language studies that included patients diagnosed with primary bone tumors located in the distal femur,
2) studies that focused on gait parameter comparisons in distal femoral resection patients, and
3) studies that presented gait analysis performed in a certified laboratory center and studies that compared stance phase, swing phase, cadence, and velocity.

#### Exclusion criteria

1) Non-English studies and unpublished studies,
2) non-comparative studies of patients undergoing distal femoral tumor resection and studies that lack data on gait analysis,
3) review, case reports, and letters to the editor, and
4) studies that lack adequate clinical data.

In this meta-analysis, the authors stated that there were no restrictions on the date of publication for the inclusion of studies. When necessary, discussions between the reviewers resolved disputes and uncertainties regarding eligibility and viability.

### Study selection and data extraction

Two authors scanned all the abstracts and titles to evaluate whether the studies assessed the questions raised by our study. Three authors of our study collected the outcomes from the studies. The authors constructed a structured table and then inputted all the data and the related information into a database. The following data were extracted from articles according to the inclusion criteria: the name of the first author, year of publication, study design and protocol, number of patients in each group, patients’ age and gender, stance phase, swing phase, velocity, and cadence.

### Quality assessment and outcome measurement

All the included studies were retrospective, and the included studies focused on similar research issues. The included studies had a low bias and disparity as studies, were similar in the context of inclusion criteria, surgical procedures, and study periods. The Newcastle–Ottawa Scale (NOS) was used for the quality assessment of the meta-analysis (14). In our study, the primary outcome was the stance phase, and the secondary outcomes were the swing phase, cadence, and velocity. Gait parameters were defined as parameters obtained after participants walked in a pathway guided by analytics, and these parameters were used to assess dynamic posture and coordination during movements.

### Statistical analysis

The measured outcomes of our study were the stance phase, swing phase, cadence, and velocity, which were all continuous data. T he software Cochrane Collaboration (ReviewManager 5.2) was used to compute the standardized mean difference (SMD) and 95% confidence intervals (CIs) for all outcomes, as the ReviewManager illustrates a more detailed forest plot in continuous data analysis. Statistical heterogeneity among the included studies was evaluated by the I^2^ tests (13). Statistically significant heterogeneity was defined as an I^2^ value >0.5 (13). Heterogeneity in our study was defined as low, moderate, and high based on I^2^ value (<40%, low; 30%–60%, moderate; 50%–90%, substantial; >75%, high). I^2^ illustrates the percentage of the total variability in effect estimates among trials that are due to heterogeneity rather than chance (13). A random-effects model was selected for heterogeneous data; otherwise, a fixed-effects model was selected. Funnel plots were used to detect the publication bias, which exhibited the intervention effect from the individual study against the respective standard error. A symmetrical inverted funnel-shaped plot suggested that there was no publication bias, and publication bias was suggested by the asymmetry of the plot.

## Results

### Study selection

In the primary article search, 112 relevant articles were retrieved, and 50 were excluded based on the exclusion criteria. The abstracts of the remaining 62 were screened, and 32 were excluded based on the exclusion criteria. After all the reviews of the remaining 30 studies, 15 were excluded due to a lack of outcome (n = 15) and duplication in the study population with other articles (n = 9). In a word, to conduct a meta-analysis, a total of six articles were included in the meta-analysis. The study selection chart is presented in **Figure 1**. The basic characteristics of the included studies are summarized in **Table 1**, and the outcomes are summarized in **Table 2**. There were 224 patients in total in these studies, and all the patients were diagnosed with primary bone tumors. Reconstruction methods used in studies were endoprosthesis, allograft, and mega prosthesis. Gait analyses were conducted at each study in different durations, but all the studies had one thing in common: each study conducted analysis after 6 months of post-operative duration and was conducted in certified laboratory centers.

**Table 1:** Characteristics of the included studies.

Studies	Study period	Time of the gait study (months)	Patient number	Male/Female	Median age	Study design	Newcastle– Ottawa Quality Score	Country
Algheshyan 2015	NA	28– 144	20	NA	26.58	Retrospective	8	USA
Benedetti 2000	1986–2009	22– 104	16	12/4	28.5	Retrospective	9	Italy
Bruns 2016	NA	12	14	13/13	33.5	Retrospective	8	Germany
De Visser 2000	NA	12– 24	19	12/7	45	Retrospective	8	Netherlands
Pellegrino 2020	2006–2016	12	26	13/13	40.9	Retrospective	8	Italy
Rompen 2015	1979–1998	6 –228	18	12/6	23	Retrospective	8	Netherlands

NA (not available) indicates that information was not available in the respective trial.

**Table 2:** Outcomes of the included studies.

Reference	Stance phase	Swing phase	Cadence Operated, healthy population	Velocity Operated, healthy population
Algheshyan 2015	5	NA	NA	NA
Benedetti 2000	9	NA	9, 10	NA
Bruns 2016	9	NA	NA	NA
De Visser 2000	10	NA	NA	9, 10
Pelligrino 2020	26	26	26, 127	26, 127
Rompen 2015	18	18	NA	NA

This denotes the number of patients with reported outcomes in the respective studies. Cadence and velocity had two different sets operated on: patients and healthy populations. T he two studies that compared healthy populations had a population of 147, and the study that compared the gait in operated and non-operated patients had a population of 77. NA (not available) indicates that information was not available in the respective trial.

### Stance phase

**Figure 2:**
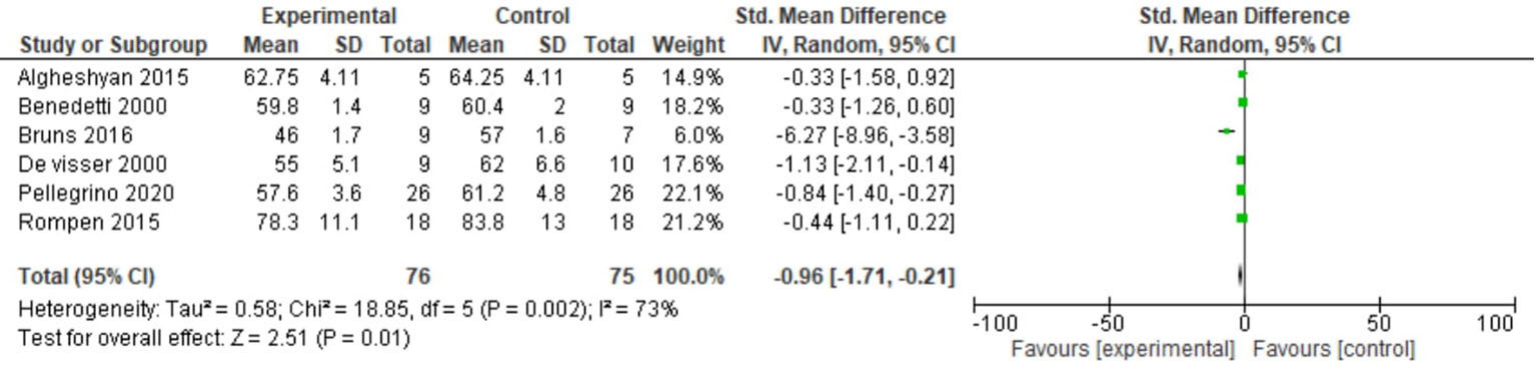
Forest plot of stance phase comparing operated and non-operated limbs of distal femur tumor patients.

The stance phase was recorded in all six studies in either the operated limbs or the non-operated limbs. For this outcome, a random-effects model analysis was used. There was a minimal decrease in SMD of the stance phase in the operated limbs than in non-operated limbs of patients undergoing distal femoral resection (SMD = −0.96, 95% CI [−1.71, −0.21], p = 0.002) as shown in **Figure 2**.

### Swing phase

**Figure 3:**
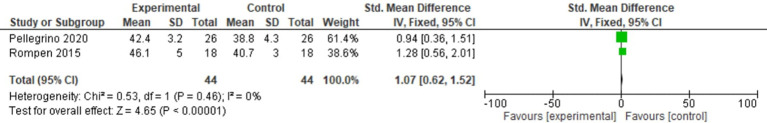
Forest plot of swing phase comparing operated and non-operated limbs of distal femoral tumor patients.

Two studies reported the swing phase. For this outcome, a fixed-effects model analysis was used. There was a minimal increase in the swing phase in the operated limbs than non-operated limbs of patients undergoing distal femoral resection (SMD = 1.07, 95% CI [0.62, 1.52], p = 0.46) as shown in **Figure 3**.

### Cadence

**Figure 4:**
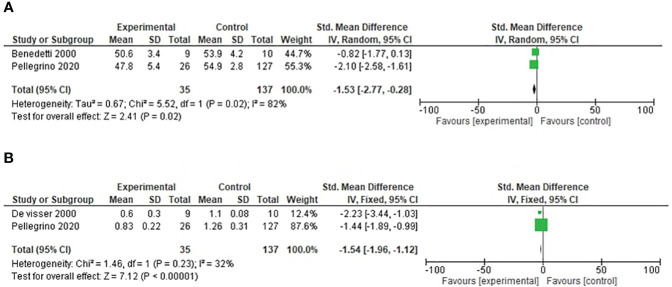
**(A)** Forest plot of cadence comparing operated and healthy populations in distal femoral tumor patients. **(B)** Forest plot of velocity comparing operated and healthy populations in distal femoral tumor patients.

Two studies reported cadence in our study. For this outcome, a random-effects model analysis was used. There was a minimal decrease in the SMD of cadence in the operated limbs compared with healthy populations in patients undergoing distal femoral resection (SMD= −1.53, 95% CI [−2.77, −0.28], p = 0.02) as shown in **Figure 4**.

### Velocity

Two studies reported velocity in our study. For this outcome, a fixed-effects model analysis was used. There was a minimal decrease in the SMD of velocity in the operated limbs compared with healthy populations in patients undergoing distal femoral resection (SMD = −1.54, 95% CI [−1.96, −1.12], p = 0.34) as shown in **Figure 4**.

### Sensitivity analysis

Sensitivity analyses were performed to determine the reliability of the results of the included studies by removing each study in turn. The magnitude and direction of the combined estimates did not change significantly by excluding individual studies, suggesting that the meta-analysis results are reliable and that the results of this meta-analysis are significant and not fluctuating. A sensitivity analysis was performed for the primary outcome stance phase. The statistical values were as follows: when the first study was excluded (SMD = −1.11, 95% CI [−1.97, −0.24], p = 0.001), when the second study was only excluded (SMD = −1.15, 95% CI [−2.06, −0.24], p = 0.001), when the third study was only excluded (SMD = −0.60, 95% CI [−1.00, −0.30], p = 0.67), when the fourth study was excluded (SMD = −0.97, 95% CI [−1.88, −0.07], p = 0.001), when the fifth study was excluded (SMD = −1.12, 95% CI [−2.17, −0.07], p = 0.0009), and when the sixth study was excluded (SMD = −1.19, 95% CI [−2.18, −0.19], p = 0.001). All the sensitivity analysis figures are added in the **Supplementary Material**.

### Publication bias

The funnel plots of the stance phase, cadence, velocity, and swing phase are shown in the figure added in the **Supplementary Material**. The funnel plot was used for all the outcomes of our study. The findings of the funnel plots stated that there was no evidence of publication bias in all the outcomes.

## Discussion

Gait analysis is one of the most important components of functional outcome assessment in patients with lower-extremity tumors (14). The gait pattern after lower-extremity limb salvage surgery is expected to be abnormal due to the amount of bone and soft tissue resection when compared to joint replacement surgery (7). Most of the reconstructed distal femurs following tumor resection yielded a reduced range of motion as reflected in observed flexion and extension when compared to normal limbs (15). Knee gait parameters are affected in distal femoral resections (14). Resected tumor patients need a high degree of knee extension for the loading affecting the stance phase (16). Knee flexion is lower resulting from weak quadriceps affecting the initial response of the gait cycle ultimately affecting the swing phase (14). Temporal parameters of gait include step time, stride time, stance time cadence, swing time, and single limb support time, and these parameters contribute to a variability domain of gait performance (17, 18). However, in our study, we could not include all these parameters, as we could not find sufficient studies comparing these parameters.

In our study, we observed a minimal decrease in SMD of the stance phase in the operated limbs than the non-operated limbs (SMD = −0.96, 95% CI [−1.71, −0.21], p = 0.002). The stance phase represents 60% of the gait cycle. Biomechanical gait characteristic variations (such as the decrease in gait speed, step length, and width; an increase in the shorter swing phase time; and a reduction of stance phase time) can be observed through kinematic gait analysis (19). These gait characteristic variations are often reported in the elderly, but the main factor related to gait performance is the reduction in muscular strength of the lower limbs, especially the quadriceps muscle (19). In a study conducted by Capanna et al., the patient undergoing distal femoral resection was found to have satisfactory results in vastus lateralis and intermedius excisions and then quadriceps excision (20). Several of the studies had similar results of reduced stance phase duration, justifying our result (21, 22). The duration of the stance phase is expected to be longer in the non-operated limbs, as this presumably is consistent with taking over part of the loading function of the operated limbs (23). The non-operated limbs had to provide support, which lasted long enough to allow the swing, ultimately leading to increased swing duration (23). Even in our study, there was an increase in the swing phase of SMD of 1.07 in operated patients.

Secondary parameters also had minimum differences in our studies, which also correlates with some of the literature (14). Velocity is the single most important component of the gait cycle, as it represents the efficacy of this means of ambulation (24). The results highlighted a reduced mean velocity in operated patients when compared with the healthy participants. This finding is consistent with the study that reported velocity after limb salvage surgery is between 64% and 88% of healthy individuals, whereas this can be justified by decreased strength due to extensive soft tissues and bone resections (21). Cadence defined as a stride per minute varies according to age. In our study, cadence in both groups was also similar. Cadence (steps/min) 54.9, velocity (m/s) 1.26, stance phase 60, and swing phase 40 are the normal limits stated in a study conducted by Pelligrono et al. (21). The result of our study showed that forest plots only had minimal reductions when compared to normal individuals, signifying that resection of the tumor did not affect a large amount in the gait parameters. The authors could not find a study stating that a rehabilitation program improves gait parameters; there was one study conducted on Parkinson’s patients where the author found improved gait parameters after 10 weeks of rehabilitation (25). All the reported studies did not mention any rehabilitation program. Each study performed gait analysis after 1 to 2 years of surgery and followed the same procedure capturing motion through Vicon cameras.

A few shortcomings and limitations of this meta-analysis should be illustrated. First, the lack of comprehensive and verified data from original studies made it difficult to adjust estimates by age, menopause, lifestyle, smoking, race, and so on, while more accurate and reliable analyses required this type of adjustment. Second, we could not post other important gait parameters such as stride time, double limb support, and step time. Third, we could not add other studies other than retrospective studies such as randomized controlled trials (RCTs) and prospective studies, which could have more clarification on this rare topic. Fourth, there were only minimal studies, so it is difficult to obtain a statistically significant result.

However, our meta-analysis also has some beneficial points. First, a systematic review of the association of gait analysis in patients with distal femoral tumors was statistically more powerful than any single study. All the studies provided significant gait data, which signified the advantage of prosthesis insertion in distal femoral resection in preserving satisfactory gait functions. Second, all included retrospective studies were of high quality and met our inclusion criteria. Third, even though the included studies were few and still produced statistically significant results, our study highlighted the importance of gait variables and the need for further studies elaborating on their impact on rehabilitation.

## Data availability statement

The original contributions presented in the study are included in the article/**Supplementary Material**. Further inquiries can be directed to the corresponding authors.

## Author contributions

Conception/design: NB, WZ, and HD. Provision of study material: NB and XF. Collection and/or assembly of data: NB and DY. Data analysis and interpretation: XF and DY. Manuscript writing: NB. Final approval of manuscript: WZ and HD. I attest to the fact that all the authors listed on the title page have read the manuscript, attest to the validity and legitimacy of the data and its interpretation, and agree to its submission to *Frontiers in Oncology - Surgical Oncology*.
