# Supplementary material for: Comparative study of gait parameters of patients undergoing distal femoral resections with non-operated and healthy limbs: a meta-analysis study
Source: Front Oncol. 2023 Sep 21;13:1089609. doi: 10.3389/fonc.2023.1089609 (PMC10552754; doi:10.3389/fonc.2023.1089609)
Supplement: Supplementary file 1 [file DataSheet_1.docx]

| **Supplementary Table 1: Newcastle– Ottawa Quality Assessment Scale for Cohort Studies** | | | | | | | | | | | | |
| --- | --- | --- | --- | --- | --- | --- | --- | --- | --- | --- | --- | --- |
| Authors | Represent-ativeness of the exposed cohort | Selection of the non exposed cohort | Ascertain-ment of exposure | Demonstra-tion that outcome not present at start | Compara-bility of cohort  (2 points) | | Assessment of Outcome | Follow-up length adequate for outcome to occur | Adequacy of Follow-up of cohorts (accounted for non-index hospitals) | Total Score  (9 points possible) | |  |
| Algheshyan 2015 | 1 | 1 | 1 | 1 | 1 | 1 | | 1 | 1 | | 8 |  |
| Benedetti 2000 | 1 | 1 | 1 | 1 | 2 | 1 | | 1 | 1 | | 9 |  |
| Bruns 2016 | 1 | 1 | 1 | 1 | 1 | 1 | | 1 | 1 | | 8 |  |
| De visser 2000 | 1 | 1 | 1 | 1 | 1 | 1 | | 1 | 1 | | 8 |  |
| Pelligrono 2020 | 1 | 1 | 1 | 1 | 1 | 1 | | 1 | 1 | | 8 |  |
| Rompen 2015 | 1 | 1 | 1 | 1 | 1 | 2 | | 1 | 1 | | 9 |  |

Supplementary figure 1: Funnel plot for publication bias

1. Stance Phase


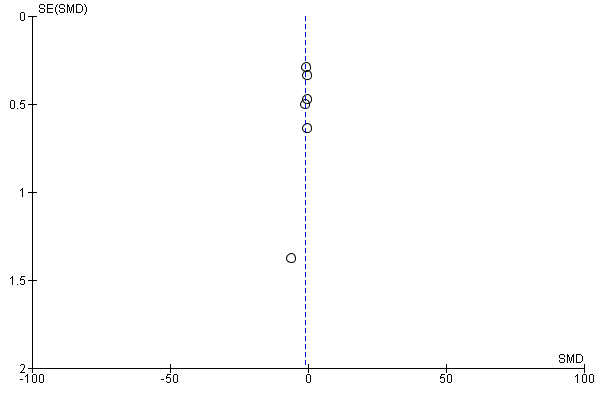


1. Swing Phase


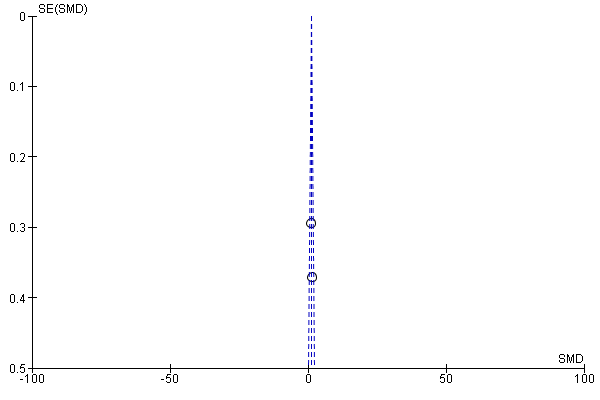


1. Cadence


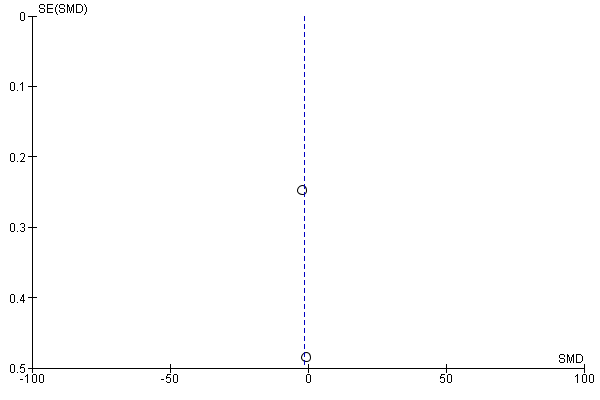


1. Velocity


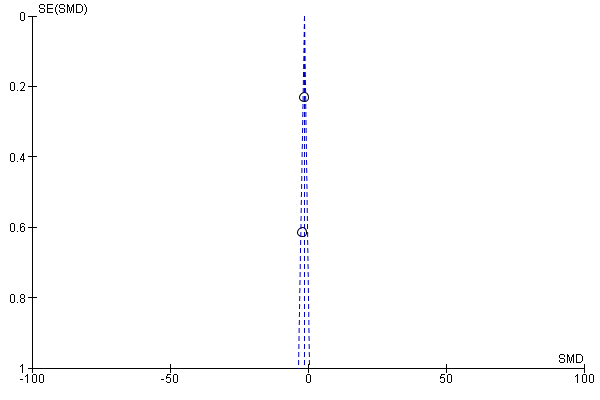


Supplementary figure 2: Results of leave-one-out sensitivity analysis


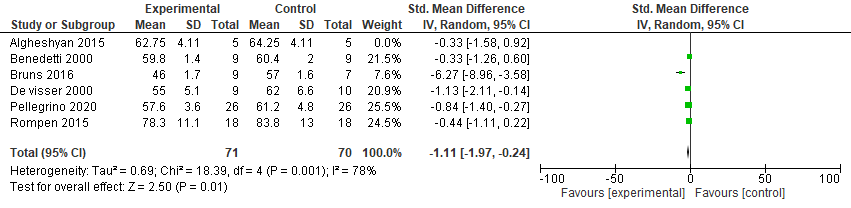


Supplementary Figure 2A: Forest plot for Stance Phase after excluding study by Algheshyan et al.


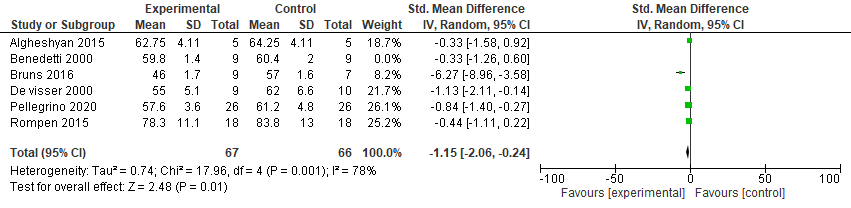


Supplementary Figure 2B: Forest plot for Stance Phase after excluding study by Benedetti et al.


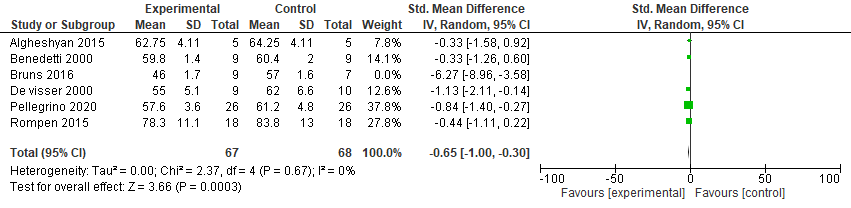


Supplementary Figure 2C: Forest plot for Stance Phase after excluding study by Huth et al.


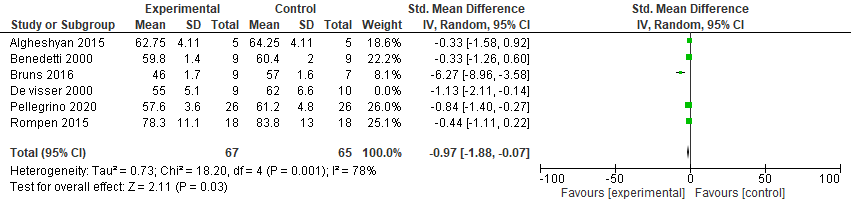


Supplementary Figure 2D: Forest plot for Stance Phase after excluding study by De visser et al.


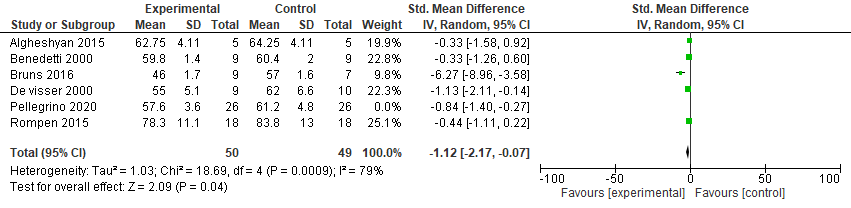


Supplementary Figure 2E: Forest plot for Stance Phase after excluding study by Pellegrino et al.


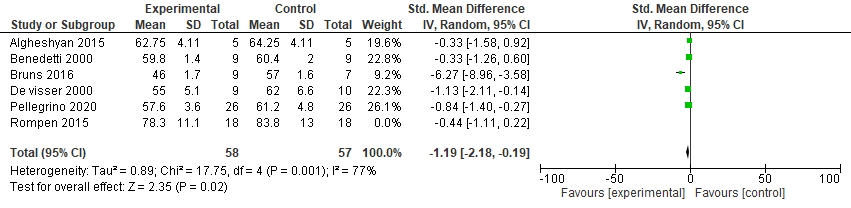


Supplementary Figure 2F: Forest plot for Stance Phase after excluding study by Rompen et al.
